# The impact of telecom industry employees’ stress perception on job burnout: moderated mediation model

**DOI:** 10.1186/s12889-024-18704-6

**Published:** 2024-06-18

**Authors:** Ruihong Liu, Hanzhong Zhang, Chunyuan Feng, Xueyi Wu, Zhenyu Pan, Wanyu Li, Liping Jia

**Affiliations:** 1 School of Psychology, Shandong Second Medical University, Weifang, China; 2 Deloitte Consulting (Shanghai) Co Ltd, Shanghai, China

**Keywords:** Telecom industry employees, Job burnout, Stress perception, Social support, Gender

## Abstract

**Background:**

The rapid development of the telecommunications industry in the post-COVID-19 era has brought tremendous pressure to employees making them a high-risk group for job burnout. However, prior research paid less attention to the burnout of employees. Furthermore, social support and gender have separate effects on job burnout. This study explores the mechanism of stress perception on job burnout and examines the roles of social support and gender amid it.

**Method:**

This cross-sectional study was conducted from June 2023 to August 2023 in mainland China. A total of 39,507 were recruited by random sampling and online questionnaires, and 28,204 valid questionnaires were retained. SPSS (version 26.0) and PROCESS (Model 4 & 7) were used for correlation analysis, mediation analysis, and mediated moderation analysis.

**Result:**

Stress perception can positively predict the level of job burnout of employees in the telecommunications industry, and social support plays a partial mediating role, accounts for 8.01% of the total effect, gender moderates the first half of the path in this mediation model. At the same pressure level, female can perceive more social support than male.

**Conclusions:**

Under high pressure background, employees’ job burnout varies depending on gender and the perception of social support. Therefore, telecommunications industry managers should adopt decompression measures and targeted social support resources for different groups.

## Introduction

Over the past 3 years, the telecommunications industry has experienced a profound reform in China. After the COVID-19 outbreak, people’s mobility was restricted, forcing them to depend on online means to accomplish many things in daily life. Therefore, there was an explosive growth of user demand in a short time and telecommunications employees were confronted with a sharp increase in workload. In addition, there was an emotional panic related to the risk of infection in their daily life. With the advent of the post-COVID-19 era, the digital economy is considered crucial for economic recovery and is developing rapidly. Telecommunications operators are closely related to people’s daily lives and play a significant role in fueling the development of the digital economy. Consequently, the Chinese telecommunications industry is concentrating on corporate transformation, technological innovation, meeting customers’ personalized demands, and enhancing market competitiveness. The manifestation of such transformations in employees is reflected in higher job requirements, stricter management systems, and continuously updated work skills and knowledge [1]. As the main force driving the development of the industry, it can be said that the working and living environments of the employees were full of challenges, and faced psychological pressure in many aspects during this period. Recently, Surveys such as that conducted in Tunisia have shown that 64.2% of the employees in a service provider center of a telecommunications had job burnout, and in 11.8% of these cases the burnout level was considered high [2]. Meanwhile, Several studies in China have also found that the stress levels of employees are on an upward trajectory [3, 4].

Stress is an important cause of job burnout. The Conservation of Resource Theory explains this association in terms of resource gains and losses. If an individual is chronically stressed and lacks available resources for emotional regulation, it can evolve into job burnout, an extreme state of stress [5, 6]. Previous studies indicated that work stress is a significant positive predictor of burnout among female employees in the telecommunications industry, and similar results exist in other occupations [7, 8]. Stress perception is the process by which an individual cognitively assesses the stress of various stimulus events in their working life [9], it is the result of the interaction between environmental stimuli and individual subjective evaluations [10, 11]. Thus, when facing the same source of stress, people may possess different subjective cognitive evaluations and developmental outcomes. It may be indicated that certain mediating variables play a role in the process through which work stress transforms into job burnout.

Social support refers to the material and emotional support that a person receives through social connections, mainly from family and important friends. It is an important resource for individuals to solve difficulties and relieve emotions. Numerous studies have shown that social support can inhibit or accelerate the transition from work stress to job burnout. A study of Chinese primary and secondary school teachers found that job stress can indirectly affect burnout through social support [12]. When teachers are provided with more opportunities for work mentoring and further training, they can effectively reduce the stress triggered by work problems, thus alleviating the level of job burnout. Also, the mediating role of social support has been confirmed in studies of professions such as policemen and nurses. So, we formulated the hypothesis 1: social support as an important resource may play an intermediary role between stress perception and job burnout.

Gender differences in the workplace have been the hot subject of extensive attention by researchers, especially the special circumstances faced by female employees. In recent years, with the continuous adjustment of the Chinese fertility policy, the family burden on women who have chosen to have a second child has increased, and their likelihood of being employed in the labor market has significantly decreased [13]. At the same time, the unfriendly labor market environment and the traditional Chinese idea of “Men outside, women inside” have caused women to experience stronger work-family conflicts than men. Studies in China and abroad have shown that women in the workplace have a higher level of stress perception [14, 15]. Therefore, gender might moderate the relationship between stress perception and job burnout.

Job burnout is a common public health problem worldwide, it not only causes physical and mental discomfort to individuals, reduces work efficiency, but also hinders social function and quality of life [2, 16–18]. However, more research on the telecommunications industry has focused on factors that improve business performance [19–21] and has lacked exploration of the underlying mechanisms by which job burnout occurs. Few studies have investigated the relationship between stress perception and job burnout and the possible influencing factors between the two within the group of telecommunications industry workers. To fill this research gap, based on the theoretical and empirical support, this study aims: (1) to investigate the extent of burnout among workers in Chinese telecommunications industry; (2) to evaluate the impact of stress perception on job burnout; (3) to explore the role of social support as mediating variable between stress perception and job burnout; (4) to explore the role of gender as moderating variable between stress perception and social support; (5) to provide theoretical and practical references for telecommunications companies to make policy adjustments and work interventions in response to the high incidence of job burnout. And the specific hypothesis, see Table 1.

**Table 1:** Study hypotheses

Hypotheses
1.Stress perception is positively correlated with job burnout
2.Stress perception is negatively correlated with social support
3.Social support is negatively correlated with job burnout
4.The relationship between stress perception and job burnout is mediated by social support
5.Gender moderated the pathway between stress perception and social support

## Materials and methods

### Research model

**Fig. 1:**
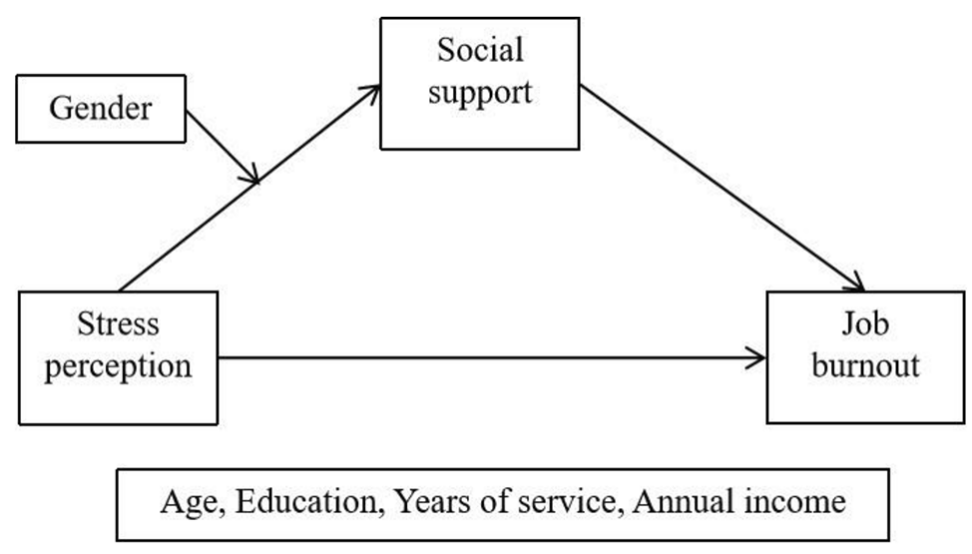
Research model

Figure 1 shows the research model proposed in this study, which examines the impact of stress perception on job burnout through the mediation of the social support and the moderation of gender.

### Study design and approach

To gain an in-depth understanding of the mental health of employees in the telecommunications industry, and to provide feasible guidance for companies to carry out work to improve their employee care mechanisms, we initiated a large-scale research project covering employees of the five major telecommunications operators in mainland China, with occupational mental health as the theme. In the preparatory stage of the research, the team set out an interview outline based on literature analysis, taking into account the development of the telecommunications industry, the characteristics of the work, and the potential psychological risks and other factors. According to the outline, the research team members interviewed 15 employees from three telecommunications operators. Based on the results of the interviews, we identified the survey themes of job burnout, stress perception, and social support, and on this basis, we selected the scales corresponding to each theme and formed the link to the electronic questionnaire. During the formal research phase, each head of the department attended training to understand the research-related issues, and then they sent the links and explained uniformly to each employee. Informed consent was obtained from each participant.

This study was conducted from June 2023 to August 2023 and was approved by the Shandong Second Medical University Ethics Committee (2023YX118). With the assistance of the All-China Federation of Trade Unions, a convenience sampling technique was employed to conduct the online questionnaire among employees of the five major telecommunications operators in twenty-two provinces, four municipalities, and five autonomous regions in mainland China. All participants completed the questionnaire anonymously by clicking on the link provided by WJX. In order to improve the validity of the collected data, the head of the department briefly explains the purpose of the research and the precautions for filling in the questionnaire to each employee. In the process of questionnaire collection, we arranged for a professional member to monitor the questionnaire filling situation in real-time and deal with sudden technical problems. At the same time, a special liaison was responsible for answering the difficulties encountered by the employees in the research process. A total of 39,507 questionnaires were collected. After eliminating based on Mahalanobis distance and answer consistency 28,204 valid questionnaires were retained. Finally, Data Processing and Statistical Analysis were performed by professional members.

### Variable measurements

#### Independent variable: stress perception

The Chinese Perceived Stress Scale (CPSS-14), translated and revised by Yang Tingzhong et al. (2003), was employed to assess the subjects’ perceived stress levels [22]. This scale consists of 14 items, categorized into two dimensions: a sense of Uncontrollability (7 items) and a sense of Tension (7 items). A 5-point Likert scoring method is used (0 = never to 4 = always). A higher score indicates a more severe perceived stress level. The Cronbach’s α values of CPSS-14 is 0.877.

#### Independent variable: job burnout

Job burnout was measured by the revised Job burnout Inventory (Maslach Burnout Inventory- General Survey, MBI-GS) translated and revised by Li Chaoping (2003) [23]. The scale includes 3 dimensions: Emotional Exhaustion (5 items), Cynicism (4 items), and Reduced Personal Accomplishment (6 items). A 7-point Likert scoring method is used (0 = never to 6 = every day). The score of each dimension is the sum of the scores of all items in this dimension. A higher score means a higher sense of job burnout. Combining the average scores of each dimension to evaluate the degree of job burnout, a score of less than 3 indicates low job burnout, a score of 3–5 indicates moderate job burnout, and a score of more than 5 indicates high job burnout [24]. The Cronbach’s α values of MBI-GS is 0.934.

#### Mediator variables: social support

Social Support Scale of the subjects was measured using the Chinese version of the social support scale translated and revised by Xie Yizhong et al. (2007) [25]. This scale has a total of 12 items, dividing the sources of social support into three main subjects: supervisor, coworker, and family member/friend (4 items each). The social support provided by each subject is divided into two types: emotional support and instrumental support (2 items each). A 5-point Likert scale is used (1 = very small to 5 = very large), with higher scores indicating more social support. The Cronbach’s α values of the scale in this study is 0.939.

#### Moderator variables: gender

A self-compiled survey including items related to age, gender, education, years of service and annual income. Sociodemographic data were examined to analyze the level of job burnout among employees in different sociodemographic trait groups, and the moderating effect of gender was also explored.

### Data analysis

Data analysis was performed using SPSS 26.0. First, we used descriptive statistics to examine the sociodemographic characteristics, stress perception, social support, and job burnout. Second, we performed a One-factor analysis of variance to identify differences in job burnout levels among groups with different sociodemographic characteristics. The statistically significant variables (*p* < 0.05) in the univariate analysis were used as adjustment variables in the subsequent model test. Third, we used Pearson’s correlation coefficients to analyze the relationships among stress perception, social support, and job burnout. Fourth, PROCESS Macro (model 4) was used to test mediating effect. When the mediating effect is significant, we then used model 7 to test the moderating effect during the mediating process. The bootstrap resampling method was applied with 5000 bootstrap samples and 95% confidence intervals (CIs). The 95% CI excluding zero indicated a significant mediating/moderating effect.

### Result

#### Common method variance

Given that the data collected in the study were all self-reported by participants, there may be issues with common method bias (CMB). CMB is the magnitude of the discrepancy between the observed relationship and the true correlation between constructs (variables) generated by common method variance (CMV) [26]. In some instances, survey participants may choose to provide answers that they perceive as socially acceptable rather than expressing their authentic views. This tendency not only impacts the simple correlations observed among the variables within a study but can also skew additional psychometric attributes of the measured constructs, such as their validity and reliability, as a result of common method variance [27]. In addition, research in 2015 showed that study indicates that the most commonly used post-hoc approach to managing CMV—Harman’s one-factor test—can detect biasing levels of CMV under conditions commonly found in survey-based marketing research [28].

Therefore, before data statistics, this study used Harman’s one factor test to examine for common method bias [29]. The results showed that exploratory factor analysis was used to extract common factors, and the amount of variation explained by the first common factor was 38.74%, which did not reach the critical standard of 40%, and subsequent research can be conducted.

### Participants’ general characteristics

Table 2 presents the descriptive statistics for specific demographic information. Female participants accounted for more than half of the study population (56.60%). Most employees are between the ages of 36 and 45 (51.60%), and hold a bachelor’s degree (71.70%). 46.60% of employees have worked for 11 to 19 years, and half of the employees (50.40%) have an annual income ranging from 50,000 to 100,000 Yuan.

**Table 2:** Demographic Information of Participants (*n* = 28,204)

Variables	Categories	Frequency	Percentage (%)
Gender	Male	12,236	43.40
	Female	15,968	56.60
Age	≤ 25	800	2.80
	26–35	6794	24.10
	36–45	14,565	51.60
	46–55	5425	19.20
	≥ 55	620	2.20
Education	Technical secondary school and below	826	2.90
	Junior college	4910	17.40
	Bachelor’s degree	20,223	71.70
	Master’s degree and above	2245	8.00
Years of service	≤ 2	984	3.50
	2–5	1702	6.00
	6–10	3116	11.00
	11–19	13,149	46.60
	≥ 20	9253	32.80
Annual income(CNY)	≤ 50,000	4727	16.80
	50,000–100,000	14,224	50.40
	100,000–150,000	6071	21.50
	150,000–200,000	1974	7.00
	≥ 200,000	1208	4.30

### Detection of Job Burnout

Among all survey respondents, telecommunications industry workers reported varying degrees of burnout. Job burnout is most common in the dimension of Reduced Personal Accomplishment with a detection rate of moderate and severe burnout of 47.07%, followed by the dimension of Emotional Exhaustion, with a detection rate of moderate and severe burnout of 29.7%, and a lower detection rate of Cynicism dimension, with 15.91% for moderate and severe burnout, see Table 3.

**Table 3:** Job burnout and detection of various dimensions among employees in the telecommunications industry

Burnout Level	Emotional Exhaustion		Cynicism		Reduced Personal Accomplishment	
	Frequency	Rate	Frequency	Rate	Frequency	Rate
Mild Burnout	20,981	74.39%	23,716	84.09%	14,929	52.93%
Moderate Burnout	5905	20.94%	3896	13.81%	12,836	45.51%
Severe Burnout	1318	4.67%	592	2.10%	439	1.56%

### Demographic differences in burnout levels among employees

One-factor analysis of variance was used to compare telecommunications industry employees with different ages, education levels, length of service, and annual income. It showed that the burnout level of employees varied between different groups, see Table 4. Analysis results as follows: the level of job burnout is slightly higher among male employees ($\stackrel{-}{\text{x}}$ = 32.90) compared to female employees. Employees aged 26 to 35 ($\stackrel{-}{\text{x}}$ = 34.30) experience more severe job burnout than those of other ages, and the problem of job burnout is more prominent among employees with junior degrees ($\stackrel{-}{\text{x}}$ = 34.17). There were two peaks of job burnout throughout the entire service life: the first is from 2 to 5 years ($\stackrel{-}{\text{x}}$ = 34.61), the second is 11 to 19 years ($\stackrel{-}{\text{x}}$ = 34.14). Furthermore, the level of job burnout tends to decrease with increasing annual income.

**Table 4:** Comparison of Burnout Levels Among Telecommunications Industry Employees Based on Different Demographic Information

Variables	Categories	Burnout Score	F/t	*p*
Gender	Male	32.90	3.401	<0.001***
	Female	32.25		
Age	≤ 25	32.85	113.983	<0.001***
	26–35	34.30		
	36–45	33.37		
	46–55	28.82		
	≥ 55	29.40		
Education	Technical secondary school and below	31.71	33.089	<0.001***
	Junior college	34.17		
	Bachelor’s degree	32.54		
	Master’s degree and above	30.29		
Years of service	≤ 2	31.86	124.058	<0.001***
	2–5	34.61		
	6–10	33.99		
	11–19	34.14		
	≥ 20	29.71		
Annual income (CNY)	≤ 50,000	35.21	150.370	<0.001***
	50,000–100,000	33.58		
	100,000–150,000	30.88		
	150,000–200,000	29.19		
	≥ 200,000	25.53		

Note. *** *P* < 0.001

### Correlation analysis between the main variables

Pearson correlation analysis was used to conduct a correlation test on job burnout, stress perception, and social support among employees in the telecommunications industry. The results show that burnout is positively correlated with perceived stress and negatively correlated with social support, and perceived stress is negatively correlated with social support. The correlation between the total scores of the three scales of job burnout, perceived stress, and social support and the scores of each dimension is shown in Table 5.

**Table 5:** Correlations (r) between job burnout, stress perception and social support (*n* = 28,204)

	$\stackrel{-}{\text{x}}$±s	1	2	3	4	5	6	7	8	9	10
1	11.71 ± 6.55	1									
2	6.74 ± 5.21	0.789**	1								
3	14.16 ± 8.32	0.255**	0.332**	1							
4	32.62 ± 15.80	0.809**	0.832**	0.742**	1						
5	11.29 ± 4.5	0.548**	0.548**	0.472**	0.657**	1					
6	12.11 ± 5.70	0.427**	0.437**	0.456**	0.561**	0.361**	1				
7	23.40 ± 8.46	0.581**	0.588**	0.559**	0.729**	0.778**	0.866**	1			
8	30.19 ± 5.94	-0.394**	-0.445**	-0.388**	-0.514**	-0.486**	-0.295**	-0.459**	1		
9	16.47 ± 3.20	-0.282**	-0.336**	-0.357**	-0.416**	-0.441**	-0.288**	-0.431**	0.638**	1	
10	46.66 ± 8.35	-0.388**	-0.445**	-0.412**	-0.525**	-0.514**	-0.320**	-0.491**	0.956**	0.837**	1

Note. ***P < 0.01; 1.Emotional exhaustion; 2. Cynicism; 3. Reduced personal accomplishment; 4. Job burnout; 5. Tension; 6. Uncontrollability; 7. Stress perception; 8. Work support; 9. Family support; 10. Social support

### Mediating Effect

In order to test the mediating effect of social support, a three-step regression analysis is performed using Model 4 of PROCESS macro. Additionally, we included control variables such as age, education, years of service, and annual income. The analysis results are presented in Table 6.

Step 1 shows the results of multiple linear regression analysis regarding the effect of the independent variable on dependent variable, stress perception was found to significantly and positively predict job burnout (*β* = 0.723, *p* < 0.01). It meant that employees with high stress perception are more likely to experience job burnout. Step 2 shows that the results of multiple linear regression analysis regarding the effect of the independent variable on mediator variable, stress perception had a negative effect on social support (*β* = -0.492, *p* < 0.01). This demonstrates that when employees are under high pressure, their perception of social support will decrease. Finally, Step 3 shows the results of analysis regarding the effects of the independent variable and mediator variables on the dependent variable, job burnout was verified to be significantly affected by stress perception (*β* = 0.615, *p* < 0.01) and social support (*β* = -0.220, *p* < 0.01). Social support plays a partial mediating role between employee stress perception and job burnout in the telecommunications industry, with the mediating effect accounting for 8.01% of the total. This shows that perceived stress can not only have a direct impact on job burnout among employees in the telecommunications industry, but also have an impact through the partial mediating role of social support.

**Table 6:** Mediating Model Test for Social Support

	Regression Equation		Overall Fit Indicators			Significance of Regression Coefficients			
	Result Variable	Predictor Variable	R	R²	*F*	*β*	SE	*t*	95%CI
Step 1	Job Burnout	Stress Perception	0.730	0.534	6452.533***	0.723	0.008	174.932	[1.334,1.365]
Step 2	Social Support	Stress Perception	0.493	0.243	1808.429***	-0.492	0.005	-93.390	[-0.495,-0.475]
Step 3	Job Burnout	Stress Perception	0.755	0.570	6235.832***	0.615	0.008	135.431	[1.131,1.164]
		Social Support				-0.220	0.008	-49.026	[-0.433,-0.400]
Total, direct, and indirect effects of X on Y									
						*β*	SE	*t*	95%CI
Total Effect						1.349	0.008	174.932	[1.334,1.365]
Direct Effect						1.148	0.008	135.431	[1.131,1.164]
Indirect Effect						0.108	0.003	/	[0.103,0.114]

Note. Covariate data etc. are omitted in the table; ***p < 0.001

### Moderating Effect

**Fig. 2:**
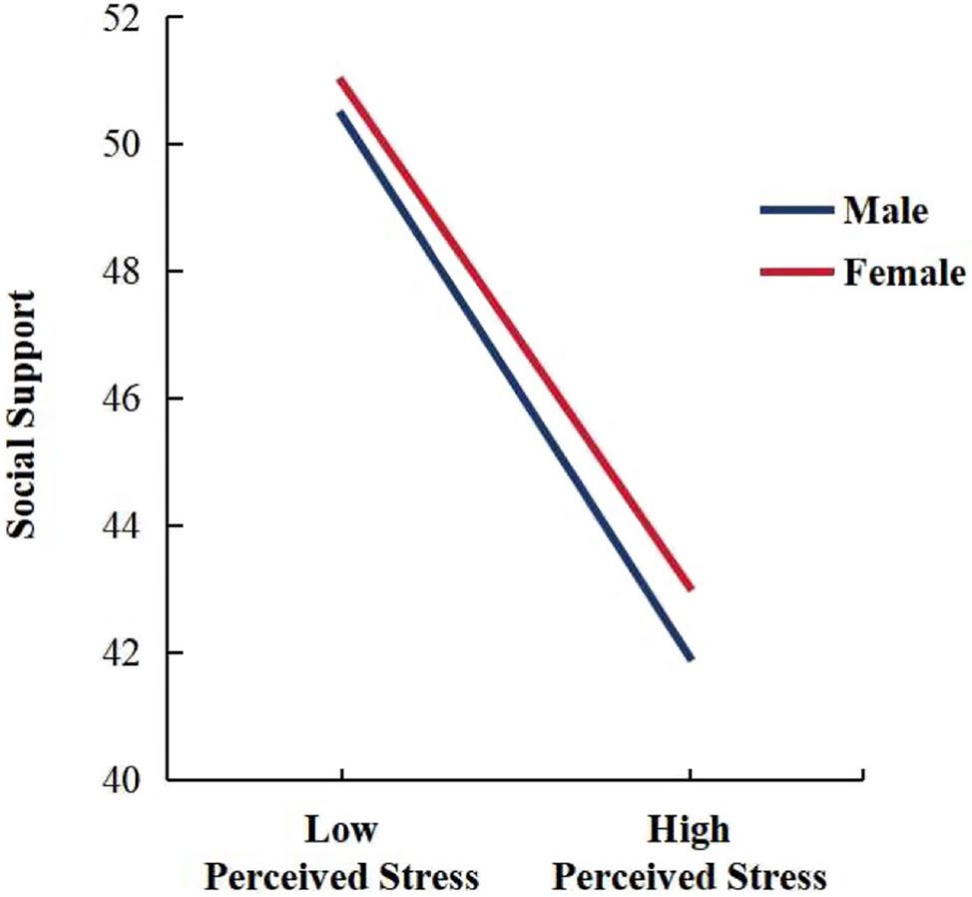
Risk Prediction Model for Perceived Stress and Job Burnout

The Model 6 of PROCESS macro was used to test the adjusted mediation model. The moderating variable was gender, and controlling the effects of age, education level, length of service, and annual income. The results show that the interaction term between stress perception and gender had a significant positive predictive effect on social support (β = 0.034, 95%CI = [0.014, 0.054]), See Table 7. It meant that gender has a significant moderating effect on the first half of the mediating pathway between stress perception and job burnout. In order to further reveal the essence of the interaction effect between stress perception and gender, male and female subjects were analyzed separately, and the interaction diagram shown in Fig. 2. As far as employees in the telecommunications industry are concerned, for female employees, stress perception has a significant negative predictive effect on social support (*β* = 0.185, 95%CI = [0.185, 0.206]), for male employees, the negative predictive effect is enhanced (*β* = 0.210, 95%CI = [0.199, 0.221]), gender playing a buffering role in the negative prediction of social support by stress perception.

**Table 7:** Risk Prediction Model of Job Burnout Based on Perceived Stress

	Y: Job Burnout			M: Social Support		
	*β*	SE	95%CI	*β*	SE	95%CI
X: Stress Perception	1.148	0.009	[1.131,1.164]	-0.504	0.008	[-0.520,-0.489]
M: Social Support	-0.416	0.009	[-0.433,-0.400]			
W: Gender				0.814	0.090	[0.638,0.990]
X*W:				0.034	0.010	[0.014,0.054]
Conditional indirect effect of X on Y of the moderator						
				*β*	SE	Boot LLCI Boot ULCI
Male				0.210	0.006	[0.199,0.221]
Female				0.196	0.005	[0.185,0.206]

Note. Covariate data etc. are omitted in the table

## Discussion

The present conducted a large-scale research on the level of job burnout among telecommunications employees in mainland China region. We also constructed a moderated mediation model to explore the mediating role of social support between stress perception and job burnout and whether gender moderated the direct relationship between stress perception and social support. As far as we know, this was the first study to analyze the path of stress perception affecting job burnout in a group of Chinese telecommunications industry, which provided theoretical support and development direction for subsequent research.

### Current status of job burnout among employees in the telecommunications industry

The results revealed that the overall job burnout was at a medium to low level, with a total score of 32.62 ± 15.80, which is lower than the average job burnout level of doctors, nurses, and police officers [30–34]. The difference in ratings may be because healthcare workers and police officers have to deal with more emergencies and more complex situations in their daily work. It makes their work more demanding, leading to higher levels of burnout induced by the workers’ higher levels of work and mental stress. By contrast, most positions of telecommunications employees have more fixed job content and less risk of responsibility at work, and the risk factors for burnout declined naturally. In addition, analyzing the burnout detection rates in each dimension, Reduced Personal Accomplishment stands out as the predominant emotional experience among employees. High job stability in the telecommunications industry can sometimes cause issues such as entrenched work patterns and limited opportunities for career advancement. These factors can contribute to a low sense of achievement among employees in this field.

Regarding the relationship between sociodemographic characteristics and job burnout, participants aged 26 to 35 with 2 to 5 years of work experience exhibit the highest levels of job burnout, showing job burnout has a young trend, which is similar to previous studies [30, 35, 36]. From the perspective of enterprise development, employees at this stage have completed their initial work adaptation, have better physical fitness and learning ability, and naturally take on more work tasks. However, they may lack sufficient work experience and career planning skills to cope with high-intensity and demanding work [37, 38]. From the perspective of family, individuals at this stage aim to build intimate relationships and raise the next generation. However, Chinese families still face indispensable financial pressures when considering childbearing decisions [39]. The simultaneous high resource consumption in both work and family domains raises the risk of burnout for individuals. It is worth noting that another peak of job burnout emerged for employees with 11 to 19 years of work experience. One possible explanation is that employees at this stage are not capable of learning enough to adapt to the transformation of the enterprise, resulting in a subjective “capability panic.” Meanwhile, this stage is the golden period for promotion, and it is hard to achieve a career breakthrough if they miss the opportunity. A combination of two factors leads to emotional imbalance and increased levels of burnout. In addition, consistent with the results of other industry studies [40–42], job burnout levels are effectively mitigated as income increases. The “Effort-Reward Model” [43] suggests that a prolonged imbalance between work investment and returns leads to job burnout, which reminds us that we should pay attention to the possible impact of employee pay distribution systems on burnout.

### The relationship between stress perception and job burnout and the mediating role of social support

The results show that employees with higher stress perception were more likely to report higher job burnout, which is consistent with previous research results [44–46]. Stressors at work can hurt the cognitive, emotional, and physiological domains of individuals. Excessive stress over a long period can deplete an individual’s energy lead to psychological disengagement from the job [47], and reduce an individual’s identification and satisfaction with the job to a certain extent [48]. Research has shown that these phenomena are crucial factors in inducing job burnout. From a neurophysiological perspective, long-term high-pressure conditions can excite the sympathetic nerve and activate the individual stress axis, which can lead to a decline in immune function, leading to depression, anxiety, and other emotional problems, contributing to job burnout [49]. Chinese telecommunications industry is facing a period of rapid development and change, a series of changes involves employees’ vital interests such as employment and benefit distribution. These changes not only raise concerns about employees’ abilities but also increase the risk of potential redundancy, causing significant emotional stress. Therefore, we can improve the status of job burnout by reducing the stress level of workers.

The results also indicated that the effect of stress perception on job burnout could be buffered by the mediating effect of social support and that both stress perception and job burnout were significantly negatively correlated with social support. Based on the Conservation of Resource Theory, When stress occurs, individuals respond in two different behavioral ways, either by immediately stopping the consumption of resources to secure the total amount of resources available or by investing a certain amount of resources to obtain a valuable return on resources to offset the losses that have happened. As mentioned earlier, the telecommunications industry is in a critical stage of development, which requires employees to complete their work with high quality. At the same time, for some positions, the degree of work completion is directly linked to the salary [50], which objectively restricts the employees from relieving the pressure by stopping the consumption of resources. As a result, employees can only keep investing in the available resources. When individuals have enough resources to supplement or get positive feedback on the resources they put in, they can maintain a balance of resources while relieving stress and preventing job burnout. On the contrary, when telecommunications workers perceive high work stress but lack social support from work and family, it will exacerbate the resource imbalance to a certain extent and increase the risk of job burnout. Indeed, the information age enables work to transcend time and space limitations, fostering a growing integration of work and family. But when work problems diffuse into the family area, work-family conflicts may occur. Therefore, individuals may have Negative cognitive bias focuses preventing them from obtaining support resources from their families, reducing the likelihood that individuals will receive effective support from the family. At the same time, employees who continue to work under high pressure may cause damage to hippocampal nerves and structural degeneration of the prefrontal cortex, leading to impairment of emotional regulation and cognitive function, which will affect an individual’s ability to use social support resources to a certain extent [51, 52]. A weakened individual’s ability to mobilize resources and reduced access to resources can result in a weakened sense of self-worth and self-confidence at work [53], driving workplace stress toward job burnout. Numerous studies on other occupations have produced consistent results [54–56], which demonstrate the cross-group stability of the mediation model constructed in this study.

### The moderating role of gender

This study examined the moderating role of gender as a moderating variable in the model. We find that gender moderates the first half of the mediating pathway of the model, gender moderates the relationship between stress perception and social support. Specifically, stress perception has a greater impact on social support for male employees. When the stress perception level is the same, male employees receive less social support than female employees. Some studies have shown that men are more independent and establish fewer prominent relationships compared to women. They tend to digest their stress internally, which makes it difficult for them to obtain social support from groups. However, women have more plentiful social circle and social support resources [57, 58]. At the same time, women are better able to acquire and transform emotional information to solve emotional problems [59]. It forcefully explains why women can better regulate and utilize social support resources under high stress perception to the effect of reducing burnout.

#### Limitations

There are some limitations of this study. Firstly, this study used a cross-sectional design, which resulted in our inability to infer a causal relationship between stress perception, gender, social support, and job burnout. Secondly, we treated employees in the telecommunications industry as a whole. Given the diverse job types within the industry, variations in levels of job burnout and its root causes could exist among different positions. Subsequent studies can cross-analyze related factors. Thirdly, the absence of a comprehensive consideration of family work pressure limits the holistic understanding of the phenomenon. To address these limitations, subsequent research should examine different job groups within the industry and incorporate the variable of family pressure. Lastly, all data we used in the study was from online questionnaires, which may affect the quality of data due to factors such as social expectations and a noisy administration environment. The validity of the study can be improved through offline questionnaires and face-to-face interviews.

### Implication

Theoretically, this study verifies the validity of the pathway of stress perception→social support→job burnout among workers in the telecommunications industry, which fills the gap of previous research in this area and provides a feasible direction and empirical support for subsequent research. Meanwhile, this study expands the scope of application of the resource conservation theory underlying this pathway in China. In addition, this study assesses the moderating role of gender, which is rare in previous studies.

The practical implications based on research results as follow:

From a prevention perspective, job burnout can be effectively prevented by reducing work stress and providing social support.

Rising work stress is an unavoidable trend in the telecommunication industry, and improving the degree of person-job matching can effectively alleviate the work stress of employees [60], which can prevent the occurrence of job burnout from the root. For an industry in transition such as the telecommunications industry, the adjustment of the job structure is inevitable, requiring the addition of new jobs on the one hand, and the adjustment of the work content of existing jobs on the other. In this context, we should clarify the work content of the existing positions and the ability of the incumbents, conduct a scientific evaluation of the ability of the current employees (such as aptitude test, emotion test, personality test, etc.), and adjust the personnel according to the evaluation results. Vacant posts should be filled through scientific recruitment, to avoid the phenomenon of employees holding multiple positions, and reduce the negative impact of role conflict.

In daily work, managers should scientifically monitor the stress level of employees and establish employee mental health files. Regularly investigate the stress level of employees, analyze the reasons for the fluctuation of the stress level of employees, and make timely adjustments to the work mode and personnel arrangements. In addition, managers should also pay attention to cultivating employees’ ability to regulate stress. Lectures and experiential activities on the subject of stress need to be provided to help employees establish a scientific understanding of stress and learn to release stress reasonably. Employees’ answers on the part of the enterprise care mechanism in the research reflect a common problem that psychological counseling work is always conducted by higher management and the usual form is preaching. Activities that lack a professional color not only blur the boundaries of the work of higher management but also fail to achieve the purpose of the activity. Therefore, managers should hire professionals to conduct activities and provide psychological counseling. Companies can also build places such as relaxation rooms and gyms to guide employees to relieve mental stress through physical relaxation.

Organizations should give adequate support resources, including work support and emotional support. On the one hand, the organization should pay attention to employees’ career planning to help them clarify their work goals and improve their motivation. On the other hand, the organization should enhance the skills of employees. Organizations should promote Mentorship, which can have a person with employees’ shortcomings of one-on-one counseling [61], to improve the employees’ learning efficiency and accomplishment. In terms of emotional support, the organization should provide employees with a complete Employee Assistance Program (EAP). Not only to solve the employee’s own problems but also to include the employee’s family in the scope of protection. This plan will not only effectively alleviate employees’ family-work conflicts, but also improve employees’ satisfaction and sense of belonging, and achieve work-family gain. It is worth noting that this study found that male workers’ ability to perceive and utilize social support is lacking relative to female workers, so when implementing employee assistance programs, organizations can consciously develop small-scale, personalized help activities for men’s strong independence and internal problem-solving characteristics.

From an intervention perspective, the problem of employee job burnout should be addressed hierarchically and logically. According to the results of this study, managers can consciously adjust the remuneration system. On the one hand, it is reflected in the balance between salary income and employee payment, and on the other hand, it is to set up a reasonable incentive mechanism. Incentive mechanisms include material incentives and spiritual incentives, for the excellent performance of the staff to arrange group trips, pay bonuses, publicize outstanding staff, and so on. In addition, managers should focus its intervention on employees aged 26–35. Firstly, strengthen the understanding of their family situation, and improve the employees’ work concentration by solving their family problems. Secondly, we should emphasize career planning guidance for these employees to help them find meaning and self-worth at work, and to facilitate the formation of internal motivation to enhance their work engagement. At the same time, for some of the employees who have worked for a little longer, we should help them to make a scientific cognition of their ability, eliminate mental fear and panic, and build up confidence in their work.

Finally, from the employees’ point of view, each employee is the person in charge and should have a clear cognition of their situation. When encountering work problems, they should take the initiative to seek help from colleagues or superiors, and they should dare to seek help from professional counselors when they feel emotionally broken. Learning to arrange work scientifically and making good use of the help provided by the organization will enable them to better adapt to the development of the industry and realize their self-worth.

## Conclusion

For all we know, this is the first study to verify the validity of the developmental mechanism by which stress perception affects job burnout through social support in a group of telecommunications employees. We have also innovatively explored the influence of gender in this mechanism. Our findings suggested that stress perception significantly predicts job burnout and can also indirectly influence job burnout through social support. In addition, gender moderated the relationship between stress perception and social support, and females were good at calling on social support resources when facing stressful situations. Therefore, managers should do an appropriate job of regulating stress among employees by carrying out activities related to stress management. At the same time, organizations should provide employees with opportunities for career development and skills training. Adopt appropriate emotional support for employees of different genders. Only when employees have sufficient resources to deal with work stress can the problem of employee burnout be effectively prevented and improved.

## Data Availability

The data and materials used in this paper are available from the corresponding author on reasonable request.
